# Attitudes Toward Older Adults: A Cross-Cultural Approach Across Residential Towns in Singapore

**DOI:** 10.1093/geroni/igaa057.3409

**Published:** 2020-12-16

**Authors:** Dylan Lien, Clara G H Chan, W Quin Yow

**Affiliations:** Singapore University of Technology and Design, Singapore, Singapore

## Abstract

Past research identified several variables that affect individuals’ attitudes toward older adults (OA), such as age and experience living with OA. However, the effect of environmental variables on these attitudes, such as ethnic-culture and proportion of OA living in their neighborhood, remain unclear. Additionally, most previous studies sampled specific populations (e.g., undergraduate students), limiting generalizability. To address these limitations, we modified the Kogan’s Attitudes Toward Old People Scale (Yen et al., 2008) and included it in a large-scale cross-sectional survey conducted among adult residents of three residential towns (MT, MaT, YT) in multiracial Singapore. The towns varied in the proportion of OA in residence, with MT having the largest proportion, YT the smallest, and MaT in between. In total, 3134 respondents completed the survey via interview. Exploratory factor analysis identified two factors: Appreciation and Prejudice. Multiple linear regressions revealed main effects of age and ethnicity, qualified by interactions of age with town, and age with ethnicity. Specifically, respondents from MaT showed greater increase in Appreciation with age compared to those from MT (t=-2.04, p=.003) and YT (t=-2.95, p=.042). There were also increases in Appreciation with age among participants of three ethnicities (Chinese, Malay, Others; t=-3.95, p<.001; no increase with age for Indian participants). Separately, there was a main effect of age on Prejudice, where Prejudice increased with age (t=4.21, p<.001). Detailed analysis will be presented to elucidate the role of environmental variables on attitudes toward OA.

